# Network pharmacology research of mechanism of Maxing Shigan Decoction in treating COVID‐19

**DOI:** 10.15302/J-QB-022-0307

**Published:** 2023-03-01

**Authors:** Xian‐Fang Wang, Zhi‐Yong Du, Qi‐Meng Li, Yi‐Feng Liu, Shao‐Hui Ma, Jui‐Wei Cui

**Affiliations:** ^1^ School of Computer Science and Technology Henan Institute of Technology Xinxiang 453003 China; ^2^ School of Sanquan Xinxiang Medical University Xinxiang 453003 China; ^3^ School of Computer and Information Engineering Henan Normal University Xinxiang 453003 China; ^4^ School of the First Clinical Xinxiang Medical University Xinxiang 453003 China

**Keywords:** network pharmacology, PPI, Maxing Shigan Decoction, COVID‐19, target

## Abstract

**Background:**

The COVID‐19 has a huge negative impact on people’s health. Traditional Chinese Medicine (TCM) has a good effect on viral pneumonia. It is of great practical significance to study its pharmacology.

**Methods:**

The ingredients and targets of each herb in Maxing Shigan Decoction which obtained from Traditional Chinese Medicine Systems Pharmacology (TCMSP) database, and the related targets of COVID‐19 were screened by GeneCards database based on the network pharmacology. Venn was used to analyze the intersection target between active ingredients and diseases. Cytoscape software was used to construct an active ingredient‐disease target network. The Protein‐Protein Interaction network was constructed by STRING database and Cytohubba was used to screen out the key targets. Gene Ontology (GO) functional enrichment analysis and KEGG pathway analysis were performed by David database.

**Results:**

In this study, a total of 134 active ingredients and 229 related targets, 198 targets of COVID‐19 and 48 common targets of drug‐disease were chosen. Enrichment items and pathways were obtained through GO and KEGG pathway analysis. The predicted active ingredients were quercetin, kaempferol, luteolin, naringenin, glycyrol, and the key targets involved IL6, MAPK3, MAPK8, CASP3, IL10, *etc*. The results showed that the active ingredients of Maxing Shigan Decoction acted on multiple targets which played roles in the treatment of COVID‐19 by regulating inflammation, immune system and other pathways.

**Conclusions:**

The main contribution of this paper is to use data to mine the principles of the treatment of COVID‐19 from the pharmacology of these prescriptions, and the results can be provided theoretical reference for medical workers.

## Abstract 2

**Author summary:** This paper studied the mechanism of a classical TCM prescription Maxing Shigan Decoction in the treatment of COVID‐19 based on network pharmacology. The main active ingredients of Maxing Shigan Decoction were obtained by analyzing the network, such as quercetin, kaempferol, luteolin, naringenin. The key targets of COVID‐19 were obtained, such as IL6, MAPK3, MAPK8, CASP3, IL10. The Gene Ontology enrichment analysis and Kyoto Encyclopedia of Genes and Genomes pathway enrichment analysis were performed. It was concluded that multiple active ingredients act on key targets through regulating inflammatory and immune‐related signaling pathways, and the mechanism of Maxing Shigan Decoction in treating COVID‐19 was expounded, which has some theoretical and practical significance.

## INTRODUCTION

Coronavirus disease 2019 (COVID‐19) caused by SARS‐CoV‐2 infection, has been rapidly spreading around the world since its emergence at the end of 2019, with a long incubation period, strong infectiousness and general susceptibility of people of all constituencies. It not only threatens people’s lives, but also has a huge impact on the country’s economic development and social stability. Clinical symptoms of COVID‐19 are fever, fatigue and dry cough, with characteristic imaging changes in the lungs, and complications such as severe acute respiratory tract infection and respiratory failure occurred in some patients [1]. Traditional Chinese Medicine (TCM) has played a huge role in the prevention of COVID‐19. National Health Commission of the People’s Republic of China and the National Administration of Traditional Chinese Medicine have recommended TCM treatment in diagnosis and treatment of COVID‐19 (3rd trial edition) [2].

Compound TCM has the characteristics of “multi‐component, multi‐path and multi‐target”, and has unique advantages compared with Western Medicine in the treatment of diseases [3]. In 1999, Shao Li firstly proposed the hypothesis that there is an interconnection between TCM and biomolecular networks. In September 2007, he constructed a research framework for TCM prescriptions based on biological networks [4]. Hopkins defined the concept of “network pharmacology” in October of the same year, the principle is to select specific nodes for multi‐target drug design through network analysis of drug‐target‐disease, thereby improving the efficiency of drug development [5]. Xianhai Li *et al*. developed the intelligent network pharmacology plat form unique for TCM by integrating and reorganizing the data from multiple resources, was applied to decipher the antidepressant mechanism of a commonly used prescription [6]. Xianhai Li *et al*. explored the mechanism of Qingfei Paidu Decoction (QFPDD) in blocking the transition of COVID‐19 patients from mild to severe stage, the QFPDD can prevent the deterioration of COVID‐19 in the following mechanisms, *i.e*. inhibiting SARS‐CoV‐2 invasion and replication, anti‐inflammatory and immune regulation, and repairing body damage, these results will be helpful for the prevention and treatment of COVID‐19 [7].

Maxing Shigan Decoction is derived from Treatise on Febrile Diseases written by Zhongjing Zhang. It is mainly used to treat externally contracted wind‐heat and heat congesting in the lung and has the effect of clearing lung heat and relieving asthma. Now it is often used for cold, upper respiratory tract infection, acute bronchitis, pneumonia and other diseases. Maxing Shigan Decoction is the basic prescription of Lianhua Qingwen Capsule, Jinhua Qinggan Granule, Qingfei Paidu Decoction and Huashi Baidu Decoction recommended in the recommended scheme [8]. Modified Maxing Shigan Decoction also had a significant effect in the treatment of SARS in 2003. From here we see that Maxing Shigan Decoction has a good application prospect in the treatment of antiviral infection. The characteristics of “multiple ingredients, multiple targets and multiple ways” of TCM are similar to network pharmacology [9]. Therefore, this article adopted the method of network pharmacology to construct the target of the intersection of the active ingredients of Maxing Shigan Decoction and COVID‐19, to study the mechanism of Maxing Shigan Decoction in the treatment of COVID‐19.

## RESULTS

### Active ingredients and targets analysis

The Traditional Chinese Medicine Systems Pharmacology (TCMSP) database was used to search the active ingredients of Mahuang, Xingren and Gancao, and the thresholds were set as follows: OB ≥ 30%, DL ≥ 0.18, as a result, 134 active ingredients were screened out from 756 chemical components.

After searching 134 active ingredients, 2429 potential targets were obtained, 253 targets were obtained after deletion of duplicates, which were mapped to the STRING database, and 229 species were finally obtained as targets of “Homo sapiens”. The number of active ingredients and targets of each herb are shown in Tab.1. There was no gypsum data in TCMSP database. In order to ensure the uniformity of data standard, this paper selected data without gypsum for experiment.

### Maxing Shigan Decoction targets and COVID‐19 targets analysis

**Figure 1:**
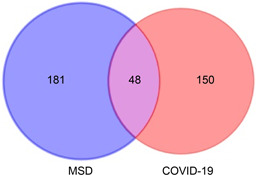
Venn diagram of Maxing Shigan Decoction (MSD) targets and COVID‐19 targets.

Took “COVID‐19” as the key word, 975 target genes related with COVID‐19 were selected from GeneCards database. With relevance score ≥ 5,198 target genes were screened and intersected with 229 targets of Maxing Shigan Decoction, and 48 targets were obtained for COVID‐19 treatment, as shown in Fig.1.

**Table 1:** The numbers of chemical components, active ingredients and targets

TCM name	Chemical components	Active ingredients	Targets
Mahuang	363	23	489
Xingren	113	19	207
Gancao	280	92	1733
Total	756	134	2429

### Active ingredients‐COVID‐19 targets network analysis

**Figure 2:**
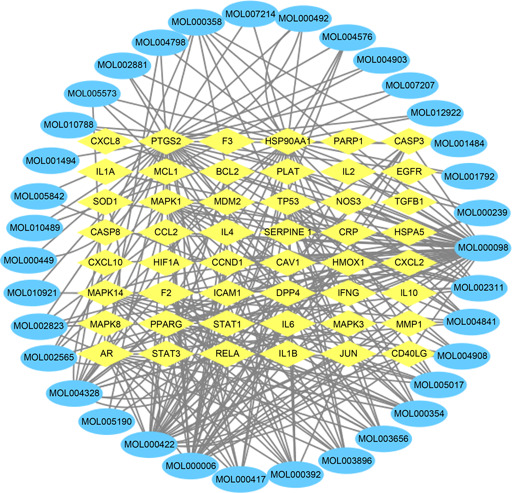
The network diagram of interaction between active components of Maxing Shigan Decoction and COVID‐19 target.

According to 134 active ingredients of Maxing Shigan Decoction and 48 intersection targets screened out by Venn diagram, the active ingredients‐targets network was constructed in Cytoscape software, as shown in Fig.2. The blue circular node represents the active ingredients, the yellow diamond node represents the targets, and the edges represents the interactions between active ingredients and targets, which fully reflect the multi‐ingredient and multi‐target characteristics of TCM. Through topology analysis of the network, the degree of each node can be obtained. The greater the degree, the more important the node is in the network. The top five active ingredients ranked by degree are shown in Tab.2.

### PPI network analysis

**Figure 3:**
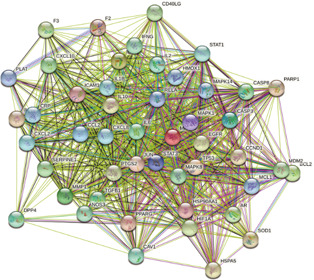
**Potential target PPI network diagram of Maxing Shigan Decoction.** The network contains 48 nodes and 749 edges.

**Figure 4:**
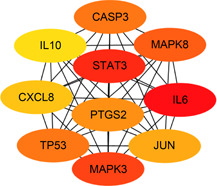
The PPI Network Diagram of key targets of Maxing Shigan Decoction

Put the targets intersection of Maxing Shigan Decoction and CIVID‐19 into STRING database, set the species as “Homo sapiens” and the confidence level as medium 0.400, PPI network was established. The network contains 48 nodes and 749 edges, as shown in Fig.3. The PPI network was imported into Cytoscape software, and ten key targets were screened in MCC (Maximum Cross Correlation) algorithm by Cytohubba plugin, including IL6, MAPK8, CASP3, IL10, PTGS2, *etc*. as shown in Fig.4.

**Table 2:** Top five active ingredients

MOL ID	English name	Degree
MOL000098	Quercetin	41
MOL000422	Kaempferol	32
MOL000006	Luteolin	22
MOL004328	Naringenin	17
MOL002311	Glycyrol	12

### GO and KEGG pathways enrichment analysis

There are 48 intersection targets which are input into the DAVID database for GO and KEGG enrichment analysis. Under the condition of *p* < 0.05 (the smaller the *p*, the higher the enrichment fraction), 94 KEGG pathways were obtained, and GO enrichment items included 308 biological processes, 31 cellular components, and 43 molecular functions.

**Figure 5:**
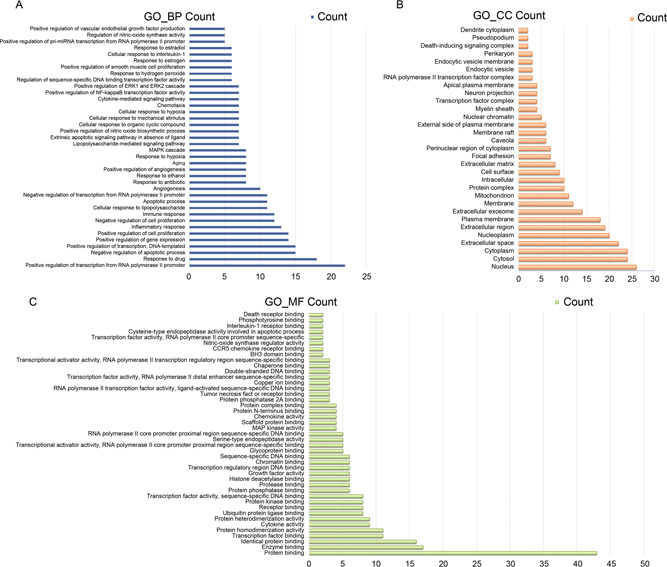
**The results of GO enrichment analysis.** (A) Biological process (BP) of the targets. (B) Cellular component (CC) of the targets. (C) Molecular function (MF) of the targets.

The results of GO enrichment analysis are shown in Fig.5. The vertical axis in the figure represents the enrichment items, while the abscissa represents the number of targets on the enrichment items. Maxing Shigan Decoction regulates biological processes by regulating genes *in vivo*. As shown in Fig.5, among the top 50 enrichment items, the most involved biological processes were positive regulation of transcription from RNA polymerase II promoter, response to drug, negative regulation of apoptotic process, positive regulation of gene expression, inflammatory response, immune response, cellular response to hypoxia, *etc*. In conclusion, Maxing Shigan Decoction treats COVID‐19 by regulating gene expression, immune system, cell response to external stimuli and other biological processes. Fig.5 shows that 31 items of cellular components include nucleus, membrane, cytosol, mitochondrion, *etc*. Fig.5 shows that there are 43 molecular function enrichment items including protein binding, enzyme binding, transcription factor binding, cytokine activity and so on.

**Figure 6:**
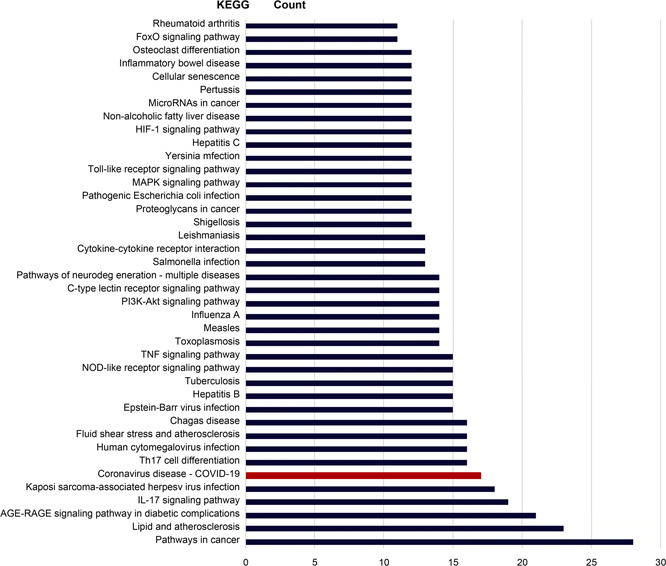
**The bar chart of KEGG channel enrichment analysis.** The vertical ordinate is the name of the KEGG metabolic pathway, and the abscissa ordinate is the number of proteins annotated to the pathway. The red legend indicates the number of genes of COVID‐19.

The results of KEGG pathway enrichment analysis are shown below in Tab.3 and Fig.6. The first 40 pathways are involved in disease‐related pathways like pathways in cancer and inflammatory bowel, infection related pathways such as influenza A, tuberculosis and herpes simplex infection, immune pathways such as T cell receptor signaling pathway, B cell receptor signaling pathway, signal transduction related pathways include the PI3K‐Akt signaling pathway, tumor necrosis factor signaling pathway, *etc*.

**Table 3:** Top 40 KEGG pathway of Maxing Shigan Decoction in treating disease

ID	KEGG	Count
hsa05200	Pathways in cancer	28
hsa05417	Lipid and atherosclerosis	23
hsa04933	AGE‐RAGE signaling pathway in diabetic complications	21
hsa04657	IL‐17 signaling pathway	19
hsa05167	Kaposi sarcoma‐associated herpesvirus infection	18
**hsa05171**	**Coronavirus disease ‐ COVID‐19**	**17**
hsa04659	Th17 cell differentiation	16
hsa05163	Human cytomegalovirus infection	16
hsa05418	Fluid shear stress and atherosclerosis	16
hsa05142	Chagas disease	16
hsa05169	Epstein‐Barr virus infection	15
hsa05161	Hepatitis B	15
hsa05152	Tuberculosis	15
hsa04621	NOD‐like receptor signaling pathway	15
hsa04668	TNF signaling pathway	15
hsa05145	Toxoplasmosis	14
hsa05162	Measles	14
hsa05164	Influenza A	14
hsa04151	PI3K‐Akt signaling pathway	14
hsa04625	C‐type lectin receptor signaling pathway	14
hsa05022	Pathways of neurodegeneration ‐ multiple diseases	14
hsa05132	Salmonella infection	13
hsa04060	Cytokine‐cytokine receptor interaction	13
hsa05140	Leishmaniasis	13
hsa05131	Shigellosis	12
hsa05205	Proteoglycans in cancer	12
hsa05130	Pathogenic *Escherichia coli* infection	12
hsa04010	MAPK signaling pathway	12
hsa04620	Toll‐like receptor signaling pathway	12
hsa05135	Yersinia infection	12
hsa05160	Hepatitis C	12
hsa04066	HIF‐1 signaling pathway	12
hsa04932	Non‐alcoholic fatty liver disease	12
hsa05206	MicroRNAs in cancer	12
hsa05133	Pertussis	12
hsa04218	Cellular senescence	12
hsa05321	Inflammatory bowel disease	12
hsa04380	Osteoclast differentiation	12
hsa04068	FoxO signaling pathway	11
hsa05323	Rheumatoid arthritis	11

**Figure 7:**
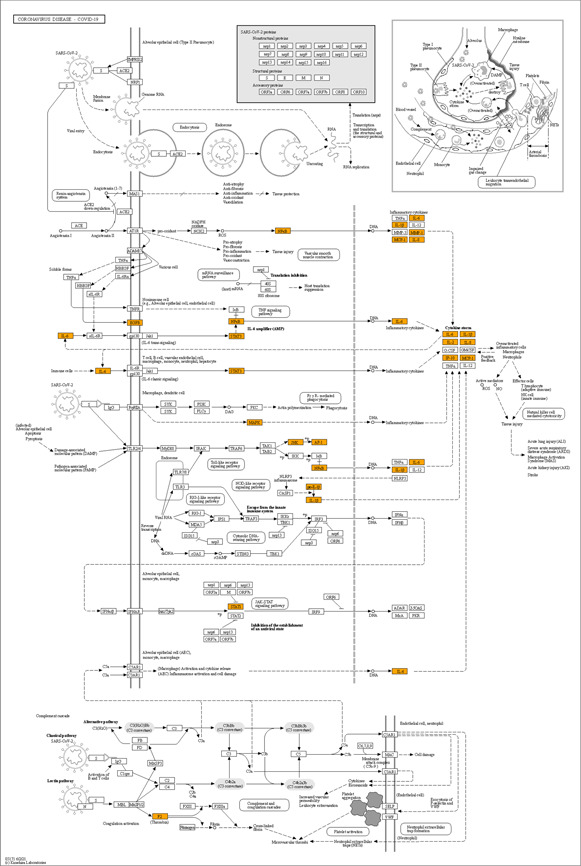
KEGG pathways of COVID‐19.

COVID‐19 is a highly contagious respiratory infection that is caused by SARS‐CoV‐2, which infects alveolar epithelial cells, mainly alveolar epithelial type 2 (AEC2) cells, through the angiotensin‐converting enzyme 2 (ACE2) receptor. Fig.7 shows that the biological process of COVID‐19 after entering the human body and the biological process involved in the common target genes of Maxing Shigan Decoction and diseases of COVID‐19, the highlight orange notes are these related target genes. Upon the occupancy of ACE2 by SARS‐CoV‐2, the increased serum level of free Angiotensin II (Ang II) due to a reduction of ACE2‐mediated degradation promotes activation of the NF‐kappa B pathway *via* Ang II type 1 receptor (AT1R), followed by interleukin‐6 (IL‐6) production. SARS‐CoV‐2 also activates the innate immune system; macrophage stimulation triggers the overproduction of pro‐inflammatory cytokines, including IL‐6, and the “cytokine storm”, which results in systemic inflammatory response syndrome and multiple organ failure. The combined effects of complement activation, dysregulated neutrophilia, endothelial injury, and hypercoagulability appear to be intertwined to drive the severe features of COVID‐19.

## DISCUSSION

Therefore, Maxing Shigan Decoction is the most frequently recommended prescription among the TCM schemes released by the state, which can treat the symptoms of COVID‐19 exosomatic wind and heat obstructing the lung [10]. Since the holistic and systematic characteristics embodied by network pharmacology are similar to the TCM theory of “holistic concept” and “treatment based on syndrome differentiation”, we adopted network pharmacology methods to study the mechanism of Maxing Shigan Decoction in the treatment of COVID‐19.

Firstly, 134 active ingredients and 229 targets of Maxing Shigan Decoction were screened out by TCMSP database, and then 198 COVID‐19 target genes were screened out by Genecards database, which were intersected with 229 targets of Maxing Shigan Decoction to obtain 48 potential targets of COVID‐19 treatment. The results of the previous step were input into Cytoscape software to construct the active ingredient‐target network, and the topology analysis of the network was carried out to obtain the active ingredients such as quercetin, kaempferol, luteolin, naringenin and glycyrol. Quercetin has the effect of anti‐regulating immunity and protecting myocardial ischemia reperfusion injury, which can effectively improve ischemia reperfusion injury caused by acute respiratory distress [11]. Kaemferol has antioxidant effect and can inhibit IL6, IL10, TNF or other genes by reducing the activity of MAKP signaling pathway. It plays an anti‐inflammatory role [12]. Naringin has anti‐inflammatory, antiviral, antitussive and immunomodulatory effects, which can protect the lung injury caused by COVID‐19. Therefore, the main active ingredients of Maxing Shigan Decoction can improve respiration and reduce inflammation. It also plays a role in the prevention and treatment of COVID‐19. Based on the PPI network of 48 targets, we screened out key targets including IL6, IL10 MAPK3, MAPK8, CASP3, PTGS2, *etc*. Studies indicate that IL plays an important role in activating and regulating immune cells and inflammation. MAPK can regulate cell growth, differentiation, stress adaptation to the environment, inflammatory response and other processes. CASP3 is also essential in the process of inflammation and apoptosis [13]. KEGG pathway analysis was performed on the targets, and 94 pathways were found to be involved in infection‐related pathways, immune‐related pathways, cancer pathways, and signal transduction related pathways. These pathways are closely associated with inflammatory response, viral infection, and immunosuppression. It is speculated that Maxing Shigan Decoction can interfere with COVID‐19 by reducing inflammation, inhibiting viruses and regulating immune system function.

## CONCLUSIONS

To sum up, this paper discussed the mechanism of Maxing Shigan Decoction in treating COVID‐19 based on the network pharmacological method. It provided ideas for the study of the treatment of COVID‐19 with TCM. There are also shortcomings in this study. One is that the lack of gypsum data has a certain impact on the prediction of active ingredients and targets, and the other is that the exact mechanism of Maxing Shigan Decoction in treating COVID‐19 still needs experimental verification.

## MATERIALS AND METHODS

### Active ingredients and targets in Maxing Shigan Decoction screening

Maxing Shigan Decoction is composed of ephedra, almond, licorice and gypsum, these components’ herb Latin names are *Ephedra sinica Stapf*, *Prunus armeniaca*, *Glycyrrhiza uralensis Fisch* and *Gypsum Fibrosum*. The chemical ingredients of Maxing Shigan Decoction were obtained by Traditional Chinese Medicine Database and Analysis Platform (TCMSP database) [14] with the keywords in phonetic transcription of “mahuang”, “xingren”, “gancao” and “shigao”. The active ingredients were selected by the conditions of oral bioavailability (OB) ≥ 30% and drug likeness (DL) ≥ 0.18 in pharmacokinetic parameters. After that the potential targets of the active ingredients of Maxing Shigan Decoction were screened out.

### COVID‐19 related targets screening

The target genes related with COVID‐19 were selected from Genecards [15] database with the keyword “novel coronavirus pneumonia”. The targets of active ingredients were intersected with COVID‐19 targets to obtain the potential targets of Maxing Shigan Decoction for COVID‐19 treatment.

### Active ingredients of COVID‐19 targets network construction

Cytoscape [16] software was used to construct a visual interaction network between the active ingredients of Maxing Shigan Decoction and COVID‐19 targets. The active ingredients and targets were represented by nodes, and the relationships between them were represented by edges.

### Protein‐protein interaction network construction

The target intersection of Maxing Shigan Decoction and COVID‐19 were imported into the STRING [17] database, and the species was set as “Homo sapiens” to obtain the protein‐protein interaction (PPI) network. Then the PPI network was imported into Cytoscape software, and the MCC algorithm was used to screen out the key targets by Cytohubba plugin.

### GO and KEGG pathways enrichment

Gene Ontology (GO) enrichment analysis and Kyoto Encyclopedia of Genes and Genomes (KEGG) pathway enrichment analysis was performed on the intersection targets using DAVID [18] software. There are three types of GO enrichment analysis, which are biological process (BP), cellular component (CC) and molecular function (MF).

### Traditional Chinese Medicine Systems Pharmacology (TCMSP) database

Traditional Chinese Medicine Systems Pharmacology (TCMSP) database is a unique systems pharmacology platform of Chinese herbal medicines that captures the relationships between drugs, targets and diseases. The database includes chemicals, targets and drug‐target networks, and associated drug‐target‐disease networks, as well as pharmacokinetic properties for natural compounds involving oral bioavailability, drug‐likeness, intestinal epithelial permeability, blood‐brain‐barrier, aqueous solubility, *etc*. This breakthrough has sparked a new interest in the search of candidate drugs in various types of traditional Chinese herbs. It can be found in ngdc.cncb website (ID:4096).

## COMPLIANCE WITH ETHICS GUIDELINES

The authors Xian‐Fang Wang, Zhi‐Yong Du, Qi‐Meng Li, Yi‐Feng Liu, Shao‐Hui Ma and Jui‐Wei Cui declare that they have no conflict of interest or financial conflicts to disclose.

All procedures performed in this study were in accordance with the ethical standards of the institution or practice at which the studies were conducted, and with the 1964 Helsinki declaration and its later amendments or comparable ethical standards.
